# Stacking orders induced direct band gap in bilayer MoSe_2_-WSe_2_ lateral heterostructures

**DOI:** 10.1038/srep31122

**Published:** 2016-08-16

**Authors:** Xiaohui Hu, Liangzhi Kou, Litao Sun

**Affiliations:** 1 SEU-FEI Nano-Pico Center, Key Lab of MEMS of Ministry of Education, Southeast University, 210096 Nanjing, China; 2 School of Chemistry, Physics and Mechanical Engineering Faculty, Queensland University of Technology, Garden Point Campus, Brisbane, QLD 4001, Australia

## Abstract

The direct band gap of monolayer semiconducting transition-metal dichalcogenides (STMDs) enables a host of new optical and electrical properties. However, bilayer STMDs are indirect band gap semiconductors, which limits its applicability for high-efficiency optoelectronic devices. Here, we report that the direct band gap can be achieved in bilayer MoSe_2_-WSe_2_ lateral heterostructures by alternating stacking orders. Specifically, when Se atoms from opposite layers are stacked directly on top of each other, AA and A’B stacked heterostructures show weaker interlayer coupling, larger interlayer distance and direct band gap. Whereas, when Se atoms from opposite layers are staggered, AA’, AB and AB’ stacked heterostructures exhibit stronger interlayer coupling, shorter interlayer distance and indirect band gap. Thus, the direct/indirect band gap can be controllable in bilayer MoSe_2_-WSe_2_ lateral heterostructures. In addition, the calculated sliding barriers indicate that the stacking orders of bilayer MoSe_2_-WSe_2_ lateral heterostructures can be easily formed by sliding one layer with respect to the other. The novel direct band gap in bilayer MoSe_2_-WSe_2_ lateral heterostructures provides possible application for high-efficiency optoelectronic devices. The results also show that the stacking order is an effective strategy to induce and tune the band gap of layered STMDs.

Compared with gapless graphene^1^,^2^, two-dimensional (2D) STMDs have attracted great interest because of its sizeable band gap^3^,^4^,^5^,^6^,^7^. Monolayer STMDs (MX_2_; M = Mo, W and X = S, Se, Te) is composed of three atomic layers, a transition-metal layer sandwiched between two chalcogen layers. Within monolayer the metals and chalcogens form strong covalent bonds, whereas in bulk STMDs, the layers are bonded by the weak van der Waals (vdW) interaction. Because of the strong intralayer interaction and the weak interlayer interaction, the ultrathin sheets of STMDs can be isolated from the bulk by the micromechanical cleavage technique^8^. The direct band gap of monolayer STMDs enables a host of new optical and electrical properties, such as strong photoluminescence^9^,^10^ and electroluminescence^11^. Novel electronic and optoelectronic devices with improved performance have also been demonstrated, such as ambipolar and high-quality field-effect transistors^12^,^13^, electric double-layer transistors^14^, integrated circuits^15^ and phototransistors with high responsivity^16^,^17^. However, experimentally it is still a challenge to precisely control layer number of STMDs, the synthesized samples are usually several layer thickness. The multilayer STMDs are indirect band gap semiconductors^9^,^10^, which limits its applicability for high-performing optoelectronic devices. If the electronic properties can be tuned from the indirect band gap to the direct band gap, they will be quite promising for practical application.

Bilayer STMDs have received much attention because they possess extra and distinct degrees of freedom, such as heterostructures and stacking orders, which makes bilayer STMDs exhibiting richer properties. Heterostructures have been the essential elements in modern semiconductor industry due to its electronic properties beyond those offered by individual constituent parts. Creating heterostructures between 2D STMDs would enable band engineering and provide new strategy to tune the electronic properties of semiconductors. Advances in 2D layered materials have allowed STMDs-based vertical and lateral heterostructures to be fabricated^18^,^19^,^20^,^21^,^22^ and meanwhile interesting physical properties have been explored on such 2D heterostructures^23^,^24^,^25^,^26^,^27^. For example, MoS_2_/WSe_2_ stacked heterostructure can form a type-II heterojunction^19^. A strong interlayer excitonic transition was observed in WS_2_/MoS_2_ vertical heterostructures^20^ and it showed a smaller band gap than the ones of WS_2_ and MoS_2_ monolayers^23^. The excellent current rectification behavior and photocurrent generation characteristic was found in WSe_2_-WS_2_ lateral heterojunctions^21^. Monolayer MoSe_2_-WSe_2_ lateral heterostructures have been achieved experimently and demonstrated enhanced photoluminescence^22^.

On the other hand, stacking faults are common in the layered materials because the energy barrier of different stacking orders is usually small, so that the sliding of layers can occur. This phenomenon has wide application in solid lubricants, such as h-BN nano-sheets^28^ or WS_2_ nanotubes^29^. The 2D nature of layered STMDs provides a unique opportunity to engineer their electronic properties by stacking monolayers in different ways^3^,^30^,^31^,^32^. Very recently, MoS_2_ bilayers with different stacking orders have been obtained by folding exfoliated monolayers experimentally, where the stacking dependent electronic properties are demonstrated^30^. In addition, Terrones *et al*. reported the possibility of direct band gap by assembling different layers of STMDs with particular stackings^31^. He *et al*. demonstrated that the electronic and optical properties of bilayer STMDs were affected by stacking orders^32^. All of these findings suggest that the heterostructure and stacking orders may offer new physical properties for layered STMDs.

Motivated by the successful fabrication and enhanced photoluminescence demonstrated in monolayer MoSe_2_-WSe_2_ lateral heterostructure^22^, we first performed first principles calculations on the electronic properties of monolayer MoSe_2_-WSe_2_ lateral heterostructure, and found the character of a direct band gap. Then we stacked additional same layer on monolayer MoSe_2_-WSe_2_ lateral heterostructures and investigated the stacking orders induced modification on the electronic properties of bilayer MoSe_2_-WSe_2_ lateral heterostructures. The direct band gap can be achieved in bilayer MoSe_2_-WSe_2_ lateral heterostructures by alternating stacking orders. Specifically, AA and A’B stacked heterostructures show weaker interlayer coupling, larger interlayer distance and direct band gap. Whereas AA’, AB and AB’ stacked heterostructures exhibit stronger interlayer coupling, shorter interlayer distance and indirect band gap. The novel direct band gap in bilayer MoSe_2_-WSe_2_ lateral heterostructures provides possible application for high-efficiency optoelectronic devices. In addition, the calculated sliding barriers indicate that stacking orders of bilayer MoSe_2_-WSe_2_ lateral heterostructures can be easily formed by sliding one layer with respect to the other. The results suggest that the feasibility of stacking orders in tuning the electronic properties of layered STMDs.

## Results

### Structural and Electronic Properties of Monolayer MoSe_2_-WSe_2_ Lateral Heterostructures

**Figure 1:**
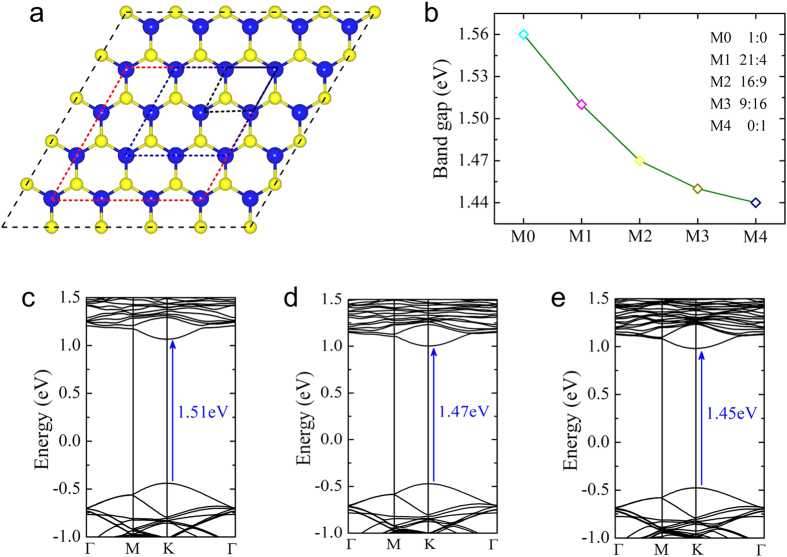
(**a**) The structural models of monolayer MoSe_2_-WSe_2_ lateral heterostructures. The dashed line rhombuses stand for the replaced MoSe_2_. The blue and yellow balls represent W and Se atoms, respectively. (**b**) The band gaps as a function of the WSe_2_ to MoSe_2_ ratio. (**c–e**) Band structures of monolayer MoSe_2_-WSe_2_ lateral heterostructures in the case of M1, M2 and M3.

In order to compare the band structures of monolayer and bilayer MoSe_2_-WSe_2_ lateral heterostructures, we first examined the lattice constants and band structures of monolayer MoSe_2_-WSe_2_ lateral heterostructures. The optimized values of lattice constants are found to be 3.316 Å (WSe_2_) and 3.319 Å (MoSe_2_), well consistent with previously reported results^33^. Because the lattice constants of WSe_2_ and MoSe_2_ are very close, monolayer MoSe_2_-WSe_2_ lateral heterostructures can be constructed simply from the primitive cells of WSe_2_ and MoSe_2_ with negligible strain. A 5 × 5 supercell of WSe_2_ is shown in Fig. 1a. Monolayer MoSe_2_-WSe_2_ lateral heterostructures can be obtained by replacing WSe_2_ with MoSe_2_ at the rhombic area. The dashed line rhombuses with different sizes stand for the replaced area. Note that the regular doping in monolayer MoSe_2_-WSe_2_ lateral heterostructures have been achieved experimently^22^, different from random doping in transition metal dichalcogenide alloys^34^,^35^. Here, we consider three monolayer MoSe_2_-WSe_2_ lateral heterostructures by varying the WSe_2_ to MoSe_2_ ratio (21:4, 16:9 and 9:16), corresponding to the small, middle and big rhombuses in Fig. 1a. For simplified, the three different ratio monolayer heterostructures are named as M1, M2 and M3, and the homogeneous WSe_2_ and MoSe_2_ are denoted as M0 and M4, shown in Fig. 1b. It is seen from Fig. 1b that the band gaps of monolayer MoSe_2_-WSe_2_ lateral heterostructures can be tuned from 1.51, 1.47 to 1.45 eV as the ratio of WSe_2_ to MoSe_2_ decreases, which are between the band gaps of homogeneous WSe_2_ (1.56 eV) and MoSe_2_ (1.44 eV). The three different WSe_2_/MoSe_2_ ratio heterostructures all exhibit a direct band gap with the valence band maximum (VBM) and conduction band minimum (CBM) located at the K point (see Fig. 1c–e). Similar to the homogeneous monolayer STMDs, monolayer MoSe_2_-WSe_2_ lateral heterostructures show the direct band gap regardless of the WSe_2_/MoSe_2_ ratio.

### Stacking Orders Induced Modification on the Electronic Properties of Bilayer MoSe_2_-WSe_2_ Lateral Heterostructures

**Figure 2:**
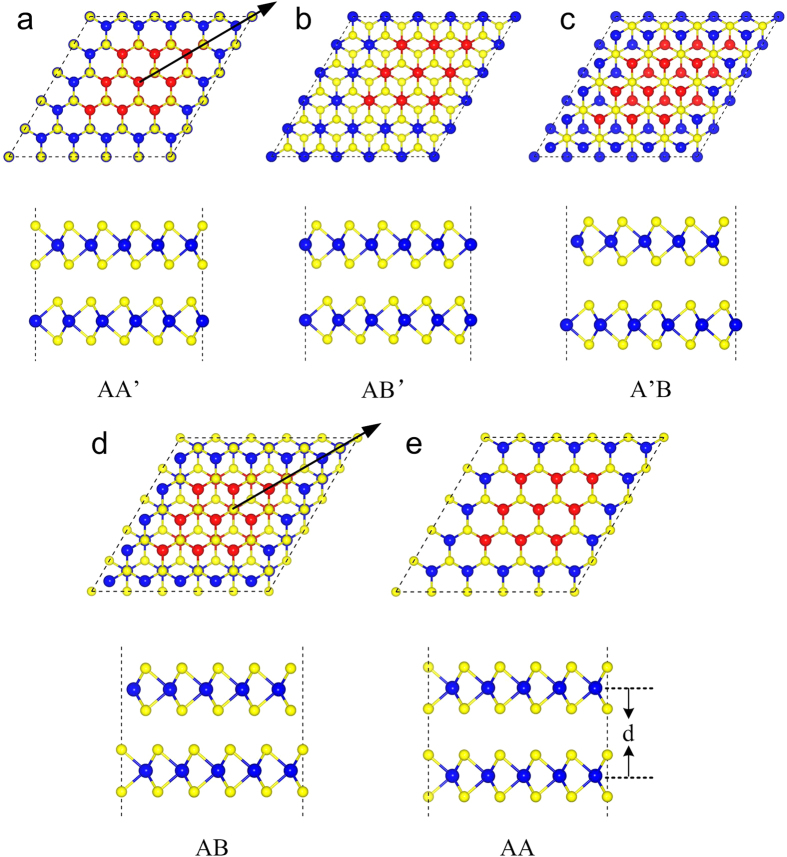
The top and side views of high-symmetry stacking orders in bilayer MoSe_2_-WSe_2_ lateral heterostructures. The arrows denote the sliding direction of the top layer in (**a,d**). The definition for the interlayer distance d is indicated in (**e**). The blue, yellow and red balls represent W, Se and Mo atoms, respectively.

The direct band gap only occurs in monolayer STMDs, the tunable electronic properties and the direct gap in bilayer STMDs are desirable. The 2D nature of layered STMDs provides a unique opportunity to engineer their electronic properties by stacking monolayers in different ways^3^. Here, we stacked additional same layer on monolayer MoSe_2_-WSe_2_ lateral heterostructures. Corresponding to the WSe_2_/MoSe_2_ ratio in monolayer MoSe_2_-WSe_2_ lateral heterostructures (M1, M2 and M3), similarly, the three different ratio bilayer MoSe_2_-WSe_2_ lateral heterostructures are named as B1, B2 and B3. The five different stacking orders in bilayer MoSe_2_-WSe_2_ lateral heterostructures are proposed. Taking B2 as an example, the five high-symmetry stacking orders are depicted in Fig. 2a–e. (i) AA’ (eclipsed with W (Mo) over Se, the characteristic of the 2H phase); (ii) AB’ (staggered with W (Mo) over W (Mo)); (iii) A’B (staggered with Se over Se); (iv) AB (staggered with Se over W (Mo), the characteristic of the 3R phase); (v) AA (eclipsed with W (Mo) over W (Mo) and Se over Se). The different stacking orders can be obtained by sliding of the top layer with respect to the bottom layer: sliding from the AA’ stacking order through the AB’ to the A’B stacking order along the arrow direction in Fig. 2a, or sliding from the AB stacking order to the AA stacking order along the arrow direction in Fig. 2d.

**Figure 3:**
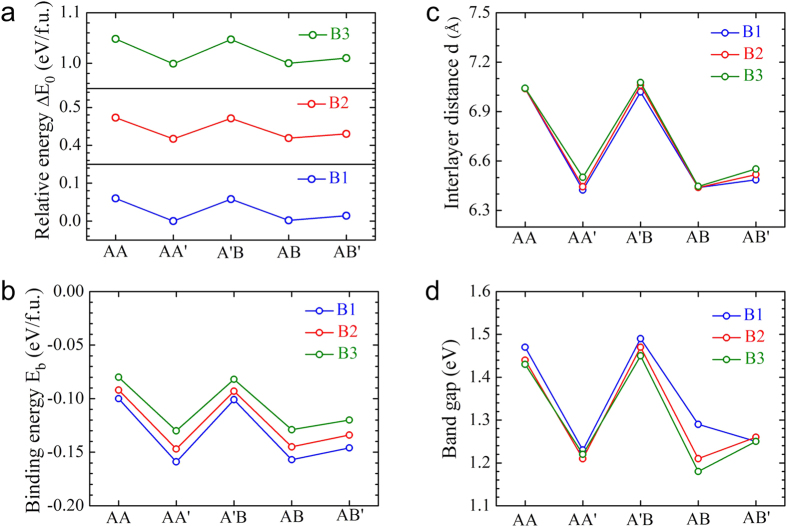
(**a**) The relative energies ΔE_0_ (eV/f.u., with respect to the lowest-energy stacking order AA’). (**b**) The binding energy E_b_ (eV/f.u.) of bilayer MoSe_2_-WSe_2_ lateral heterostructures. (**c**) The optimized interlayer distance d (Å). (**d**) Band gaps of bilayer MoSe_2_-WSe_2_ lateral heterostructures in the different stacking orders.

The relative stability of bilayer MoSe_2_-WSe_2_ lateral heterostructures was estimated and compared. It is evident from the relative energies of Fig. 3a that the three bilayer MoSe_2_-WSe_2_ lateral heterostructures exhibit a very similar behavior in the different stacking orders. Taking the case of B2 as an example, the lowest-energy stacking order is AA’, closely followed by the AB stacking, the latter is only by 2 meV per formula unit (f.u.) less stable than the former. The next stable stacking order is AB’, by 13 meV/f.u. less stable than the AA’ stacking. The remaining other two high-symmetry stackings, AA and A’B, are significantly less stable, which are 56 or 54 meV/f.u. higher than AA’ stacking, respectively. The relative stability of the other WSe_2_/MoSe_2_ ratio bilayer heterostructures (B1 and B3 cases) has the same trend.

In order to further confirm the stability of these systems, we calculated the binding energies of bilayer MoSe_2_-WSe_2_ lateral heterostructures to quantitatively characterize the binding strength between interlayer. The binding energy is defined as the energy difference between bilayer heterostructures and the corresponding monolayers (E_b_ = E_bi_ − E_mono1_ − E_mono2_). Results shown in Fig. 3b reveal that the binding energy of bilayer MoSe_2_-WSe_2_ lateral heterostructures strongly depends on the stacking orders. It is clear that the binding of AA’ and AB stacked heterostructures are obviously stronger than that of the other stacked ones. Comparing the structures in Fig. 2a,d, W (Mo) and Se atoms from opposite layers sit on top of each other in both AA’ and AB stacked heterostructures, leading to stronger interlayer coupling. Among the others, A’B and AA stacked heterostructures (Fig. 2c,e) have negatively charged Se atoms stacked directly on top of each other. By Coulomb repulsion, their interlayer distance is enlarged and the coupling is reduced. It is found from Fig. 3c that the interlayer distance d is significantly larger in AA (7.04 Å) and A’B (7.06 Å) stacked heterostructures compared to AA’ (6.44 Å) and AB (6.44 Å) stacked ones. Accordingly, the interlayer coupling strength of the different stacked heterostructures is AA’≈ AB > AB’ > AA ≈ A’B, depending on the relative atomic positions of the top layer with respect to the bottom layer.

**Figure 4:**
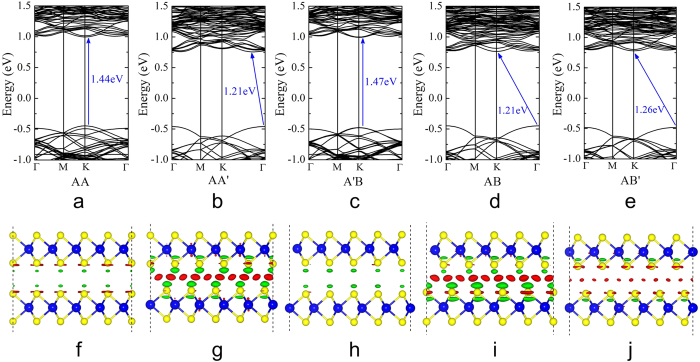
Band structures (**a–e**) and charge transfer (**f–j**) of AA, AA’, A’B, AB, and AB’ stacked bilayer MoSe_2_-WSe_2_ lateral heterostructures in the case of B2. The isosurface value for all of the cases is 2 × 10^−4^ e/Å^3^. The red isosurface indicates an electron gain, while the green one represents an electron loss.

The different stacking orders and interlayer interaction are expected to have significant effect on the electronic properties of bilayer MoSe_2_-WSe_2_ lateral heterostructures. As shown in Fig. 3d, the band gaps of bilayer MoSe_2_-WSe_2_ lateral heterostructures are quite insensitive to the WSe_2_/MoSe_2_ ratio. However, we can see the obvious role of the stacking orders on their electronic properties. The band gap values of AA and A’B stacked heterostructures are significantly larger compared with those of the other stacked heterostructures (see Fig. 3d). This intriguing electronic modulation arises from the larger difference of interlayer distances induced by the different stacking orders. Taking the case of B2 as an example, band structures of the different stacked heterostructures are compared in Fig. 4a–e. It can be classified to two types: (i) AA and A’B stacked heterostructures. It is interesting to note that a direct gap behavior at the K point is observed for AA and A’B stacking orders in bilayer MoSe_2_-WSe_2_ lateral heterostructures. The direct gap changes very little, from 1.44 eV (AA stacking) to 1.47 eV (A’B stacking), which are indicated by blue arrows in Fig. 4a,c. The direct gap behavior here is distinctive from the indirect gap in the homogeneous bilayer WSe_2_ or MoSe_2_, thus widening the possibilities of applications for bilayer STMDs in the optoelectronic area. (ii) AA’, AB and AB’ stacked heterostructures. The indirect gap behavior is obtained for AA’, AB and AB’ stacking orders. The band gaps changes from 1.21 eV (AA’ and AB stackings, see Fig. 4b,d) to 1.26 eV (AB’ stacking, see Fig. 4e). The VBM is located at the Γ point, whereas the CBM switches from T point (the midpoint between Γ and K) to the K point, resulting in different types of indirect band gaps.

From the above analysis, we can summarize the five stacking orders of bilayer MoSe_2_-WSe_2_ lateral heterostructures into two classes from the interlayer coupling, binding energies and electronic properties, namely class I (AA and A’B stacking orders) and class II (AA’, AB and AB’ stacking orders). Class I has weaker interlayer coupling/larger interlayer distance and direct band gap, whereas class II has stronger interlayer coupling, shorter interlayer distance and indirect band gap. In addition, in stark contrast, the band gap of class I is larger than that of class II. The origin of the difference is due to the larger interlayer distances in class I as compared to class II, which are d = 7.04 Å and 6.44 Å for AA and AA’ stacked heterostructures respectively (see Fig. 3c). These conclusions can be further supported by the charge transfer, which is defined as the difference of the total charge of bilayer heterostructures and two monolayers (Δρ = ρ_tot_ − ρ_mono1_ − ρ_mono2_), as shown in Fig. 4(f–j). As expected, for AA and A’B stacked heterostructures, the small amount of charge transfer was observed, indicating the weak interaction between two monolayers. In comparison to the band gap (1.47 eV) of the corresponding monolayer MoSe_2_-WSe_2_ lateral heterostructures (see Fig. 1d), the band gap of AA (1.44 eV) and A’B (1.47 eV) stacked heterostructures are almost completely unaffected. As for AA’, AB and AB’ stacked heterostructures, increasing charge transfer happens, suggesting an enhanced interaction between two monolayers, which essentially causes the band gap decrease. The same trends are also observed in the other ratio bilayer MoSe_2_-WSe_2_ lateral heterostructures (see the Supporting Information Figures S1 and S2).

**Figure 5:**
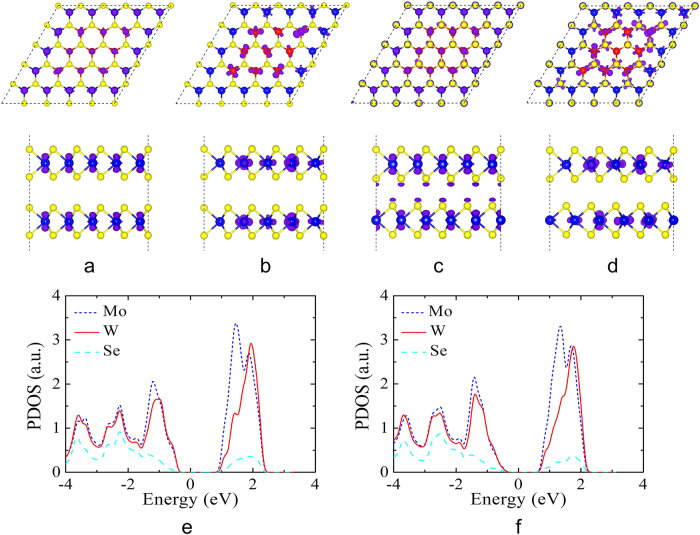
The charge densities of (**a**) VBM, (**b**) CBM, (**e**) PDOS of AA stacked, and (**c**) VBM, (**d**) CBM, (**f**) PDOS of AA’ stacked bilayer MoSe_2_-WSe_2_ lateral heterostructures in the case of B2. The isosurface value is set to be 0.001e/Å^3^.

In order to get insight into the origin of the direct and indirect band gap in different stacked heterostructures, we analyze deeply the charge distribution of the VBM and CBM states at the K point of the AA (class I) and AA’ (class II) stacked heterostructures. We present in Fig. 5 the charge densities of the VBM and CBM states and the partial density of states (PDOS) for AA and AA’ stacked heterostructures. For AA stacked heterostructure, the results indicate that the VBM state is dominated by the state from the W and Mo atoms, which are distributed throughout the whole heterostructure, see Fig. 5a. In sharp contrast, the CBM state only originates from the Mo atoms and localized on the MoSe_2_ part, see Fig. 5b. The analysis is further demonstrated by the PDOS of the W and Mo atoms in AA stacked heterostructure, as shown in Fig. 5e. It is clear that the VBM state is due to the W and Mo atoms, but the CBM state is owed to the Mo atoms. The physical information from PDOS is consistent with those from charge density distribution, which further verify our conclusion. For AA’ stacked heterostructure, it is found that the charge density distribution is very similar to those of AA stacked heterostructure. But from side view of the charge distribution, some obvious difference between AA and AA’ stacked heterostructures can be observed. There is no charge density overlap in AA stacked heterostructure (see Fig. 5a) due to its larger interlayer distance (7.04 Å). The large interlayer distance of AA stacked heterostructure leads to the weak coupling/interaction between the two monolayers, so the band structure of AA stacked heterostructure can be simply regarded as the superposition of these two monolayers. The conclusion can be verified when we compare the band structure in Fig. 1d with that in Fig. 4a. However, the distinct charge density overlap between the interlayer is found in AA’ stacked heterostructure (see Fig. 5c) because of its smaller interlayer distance (6.44 Å), which results in the strong interlayer interaction. That leads to the energy variation of the VBM state (K point) (see Fig. 4b), so the direct band gap transfers into the indirect band gap from AA to AA’ stacked heterostructures. Our results reveal the physical reasons of the direct and indirect band gap for the different stacking orders in bilayer MoSe_2_-WSe_2_ lateral heterostructures. Here, the controllable direct/indirect band gap provides new opportunities for engineering the electronic properties of bilayer STMDs. Moreover, the novel direct band gap in bilayer MoSe_2_-WSe_2_ lateral heterostructures can be used to achieve the strong photoluminescence and provides possible application in optoelectronic devices.

### Sliding Barrier of Different Stacking Orders in Bilayer MoSe_2_-WSe_2_ Lateral Heterostructures

**Figure 6:**
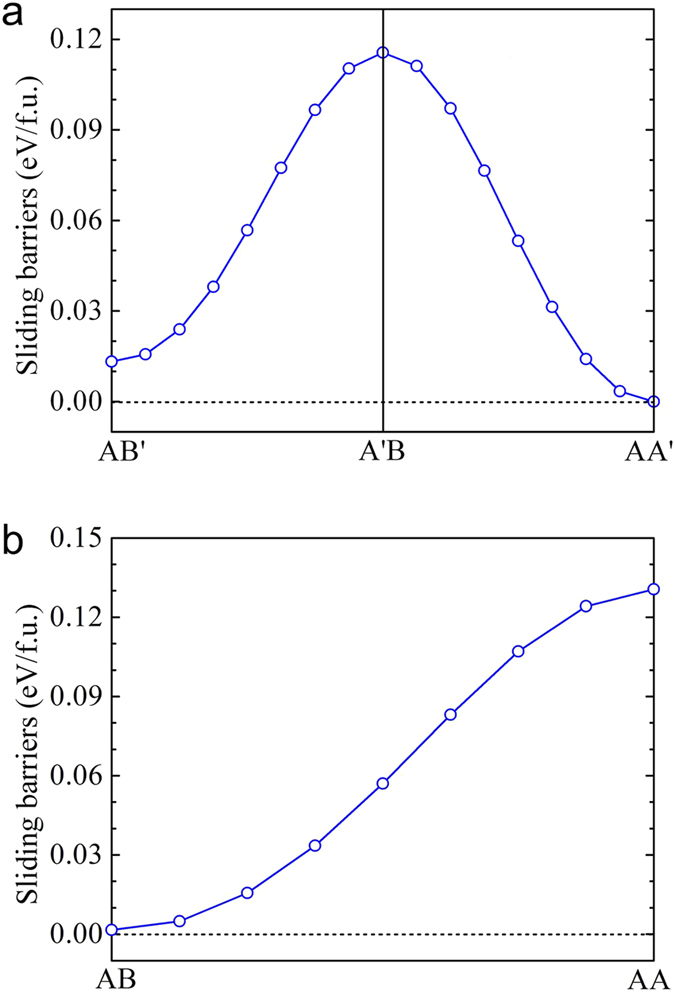
The sliding barriers of the different stacked bilayer MoSe_2_-WSe_2_ lateral heterostructures in the case of B2. The total energies of AA’ stacking are set to be zero as references, marked by a dotted line.

The above results demonstrated that the structural stability of bilayer MoSe_2_-WSe_2_ lateral heterostructures are significantly sensitive to the stacking orders and their electronic properties can widely be tuned from direct to indirect band gap. The weak vdW interaction between interlayer makes it susceptible to stacking in the layered materials. The energy barrier of different stacking orders is usually small, so that the sliding of one layer with respect to the other can occur. In order to understand the sliding behavior in bilayer MoSe_2_-WSe_2_ lateral heterostructures, taking the case of B2 as an example, we further carried out the sliding barrier calculations for bilayer MoSe_2_-WSe_2_ lateral heterostructures. The sliding barriers were evaluated using the same approach for bilayer BN^36^,^37^ and MoX_2_ (X = S, Se, Te)^38^. A set of lateral shifts of one layer with respect to the other layer were performed. At each shifted configuration we calculate the total energy of the bilayer heterostructures. Note that the interlayer distance is fixed at 6.44 Å in this set of calculations, starting from the low-energy stacking orders (AA’ and AB). The calculated sliding barriers between different stacking orders are 0.10 eV, 0.12 eV, 0.13 eV per formula unit from AB’ to A’B, AA’ to A’B, AB to AA, respectively, presented in Fig. 6. The result demonstrated that the sliding barrier of different stacking orders is small enough, which indicates that stacking orders of bilayer MoSe_2_-WSe_2_ lateral heterostructures can be easily formed by sliding one layer with respect to the other. Experimentally, bilayer MoSe_2_-WSe_2_ lateral heterostructures with AA’, AB and AB’ stackings are possible and observed in the bilayer regions of the samples. While less stable AA and A’B stackings can be obtained by folding monolayer or sliding one layer with respect to the other artificially. The recent experimental work^30^ has shown that MoS_2_ bilayers with different stacking orders can be produced by folding MoS_2_ monolayer. Our results provide a feasible strategy to obtain artificial STMDs bilayers by sliding.

## Discussion

We investigated the stacking orders induced modification on the electronic properties of bilayer MoSe_2_-WSe_2_ lateral heterostructures. The direct band gap can be achieved in bilayer MoSe_2_-WSe_2_ lateral heterostructures by alternating stacking orders. Specifically, the five stacking orders of bilayer MoSe_2_-WSe_2_ lateral heterostructures can be summarized into class I (AA and A’B stacking orders) and class II (AA’, AB and AB’ stacking orders) from the interlayer coupling, binding energies and electronic properties. Class I shows weaker interlayer coupling, larger interlayer distance and direct band gap, whereas class II exhibits stronger interlayer coupling, shorter interlayer distance and indirect band gap. The results elucidated the importance of stacking orders in controllably tuning the band gap of bilayer MoSe_2_-WSe_2_ lateral heterostructures, suggesting that the stacking orders is an effective strategy to induce and tune the band gap of layered STMDs. The novel direct band gap in bilayer MoSe_2_-WSe_2_ lateral heterostructures provides a promising candidate for applications in optoelectronic devices.

## Methods

Our calculations were performed using plane-wave density functional theory (DFT), as implemented in the VASP package^39^,^40^. The projector-augmented-wave (PAW) potentials were used to account electron-ion interactions^41^,^42^, while the electron exchange-correlation interactions were treated using generalized gradient approximation (GGA) in the scheme of Perdew-Burke-Ernzerhof (PBE)^43^. Van der Waals corrections were included through Grimme’s DFT-D2 method as implemented in VASP^44^,^45^. A kinetic energy cutoff was set to 500 eV. The atomic positions were relaxed until forces were converged to less than 0.02 eV/Å. A k-point sampling of 9 × 9 × 1 was used for the structural relaxations, and a vacuum region of 17 Å was introduced to avoid interaction between periodic images of slabs.

## Additional Information

**How to cite this article**: Hu, X. *et al*. Stacking orders induced direct band gap in bilayer MoSe_2_-WSe_2_ lateral heterostructures. *Sci. Rep.* **6**, 31122; doi: 10.1038/srep31122 (2016).

## Supplementary Material

Supplementary Information
