# Supplementary material for: Stacking orders induced direct band gap in bilayer MoSe2-WSe2 lateral heterostructures
Source: Sci Rep. 2016 Aug 16;6:31122. doi: 10.1038/srep31122 (PMC4985822; doi:10.1038/srep31122)
Supplement: Supplementary Information [file srep31122-s1.pdf]

Stacking orders induced direct band gap in bilayer $MoSe_2$-WSe_2 lateral heterostructures

Xiaohui Hu[1], Liangzhi Kou[2]* & Litao Sun[1]*

[1]SEU-FEI Nano-Pico Center, Key Lab of MEMS of Ministry of Education, Southeast University, 210096 Nanjing, China

[2]School of Chemistry, Physics and Mechanical Engineering Faculty, Queensland University of Technology, Garden Point Campus, Brisbane, QLD 4001, Australia

*Correspondence to: L.S. (slt@seu.edu.cn) or L.K. (kouliangzhi@gmail.com)

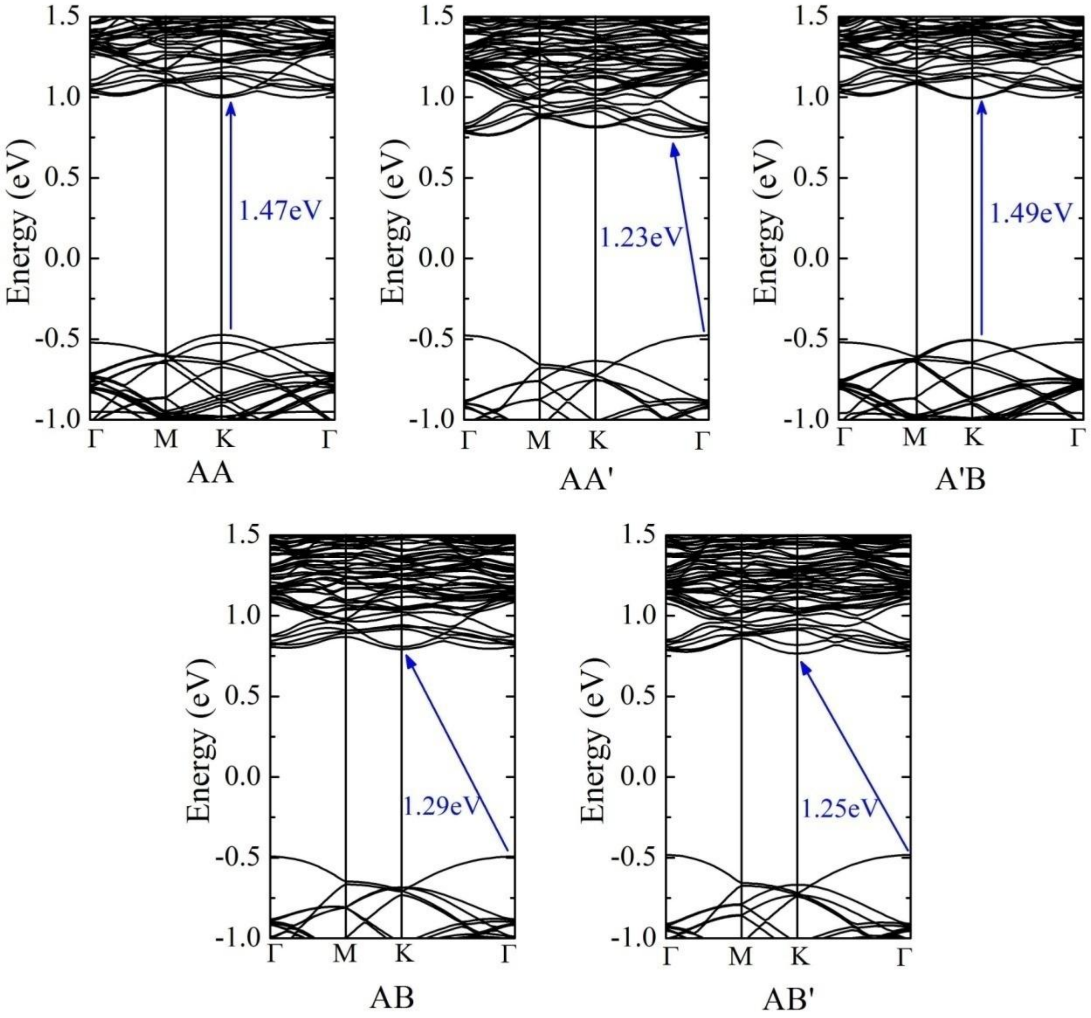

Figure S1 Band structures of AA, AA’, A’B, AB, and AB’ stacked bilayer $MoSe_2$-WSe_2 lateral heterostructures in the case of B1.

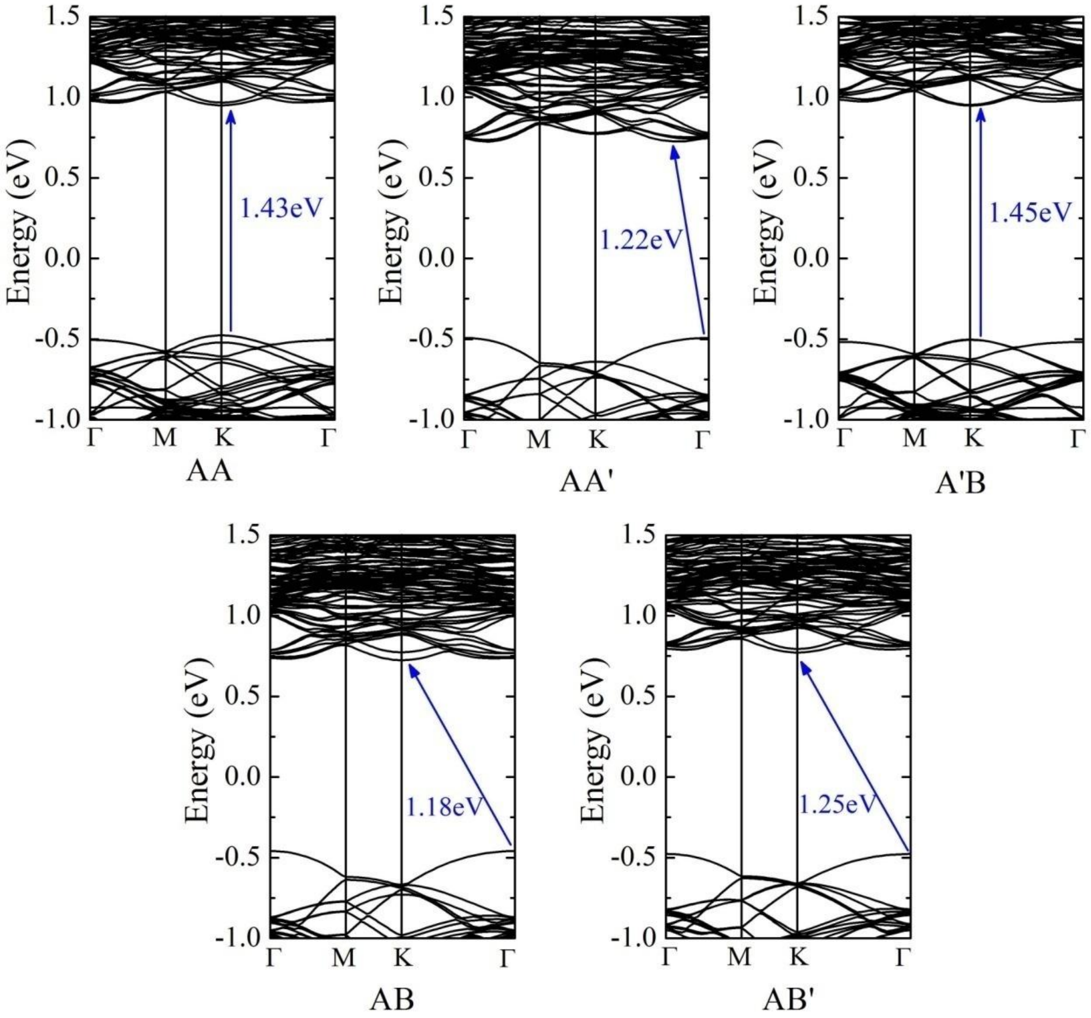

Figure S2 Band structures of AA, AA’, A’B, AB, and AB’ stacked bilayer $MoSe_2$-WSe_2 lateral heterostructures in the case of B3.
